# An Encryption Algorithm for Region of Interest in Medical DICOM Based on One-Dimensional *e^λ^*-cos-cot Map

**DOI:** 10.3390/e24070901

**Published:** 2022-06-29

**Authors:** Xin Meng, Jinqing Li, Xiaoqiang Di, Yaohui Sheng, Donghua Jiang

**Affiliations:** 1 School of Computer Science and Technology, Changchun University of Science and Technology, Changchun 130022, China; mengxin97@mails.cust.edu.cn (X.M.); dixiaoqiang@cust.edu.cn (X.D.); syh@mails.cust.edu.cn (Y.S.); 2 Jilin Province Key Laboratory of Network and Information Security, Changchun 130033, China; 3 Information Center, Changchun University of Science and Technology, Changchun 130022, China; 4 School of Computer Science and Engineering, Sun Yat-sen University, Guangzhou 511400, China; jiangdonghua@chd.edu.cn

**Keywords:** 1D *e^λ^*-cos-cot map, chaotic performance, medical image encryption, region of interest, selective image encryption

## Abstract

Today, with the rapid development of the Internet, improving image security becomes more and more important. To improve image encryption efficiency, a novel region of interest (ROI) encryption algorithm based on a chaotic system was proposed. First, a new 1D $e^{\lambda }$-cos-cot (1D-ECC) with better chaotic performance than the traditional chaotic system is proposed. Second, the chaotic system is used to generate a plaintext-relate keystream based on the label information of a medical image DICOM (Digital Imaging and Communications in Medicine) file, the medical image is segmented using an adaptive threshold, and the segmented region of interest is encrypted. The encryption process is divided into two stages: scrambling and diffusion. In the scrambling stage, helical scanning and index scrambling are combined to scramble. In the diffusion stage, two-dimensional bi-directional diffusion is adopted, that is, the image is bi-directionally diffused row by column to make image security better. The algorithm offers good encryption speed and security performance, according to simulation results and security analysis.

## 1. Introduction

With the rapid development of information and Internet technologies, a large amount of information needs to be transmitted over the Internet, so information security has received wide attention. Digital images, as an important data content for multimedia transmission, contain a large amount of private visual information, and their secure transmission in public channels and trusted storage in cloud environments are difficult to fully guarantee [1]. For some special fields, such as military, business, and medicine, digital images have stricter requirements for their confidentiality. As a special type of image, medical images are different from ordinary digital images, carrying important information closely related to patients’ lives and having strong sensitivity and privacy. The leakage and destruction of medical images during transmission in today’s more advanced telemedicine may cause significant damage and harm to medical institutions and individual patients, and may even constitute a threat to health and life. To facilitate the sharing and remote processing of medical images in a more secure way, many people have tried different methods, the most common ones include image encryption [2,3,4], data hiding [5,6], and image watermarking [7,8]. Among these methods, image encryption is considered to be one of the most important methods for securing image data [9].

Medical images cannot match the requirements of real-time transmission of current information because they contain a lot of data, have a slow encryption speed, and take a long time to encrypt. As a result, traditional encryption algorithms such as AES and DES [10,11,12] are no longer suited for the encryption of medical images. Many researchers have discovered that chaotic systems are characterized by ergodicity, unpredictability, and sensitivity to beginning values, which makes chaos well suited for picture encryption [13,14,15]. Low-dimensional chaotic systems and high-dimensional chaotic systems are the most common classifications for chaotic systems. Refs. [16,17] proposed a method of making a simple and effective chaotic system by using the difference of the output sequences of two identical existing one-dimension (1D) chaotic maps. Simulations and performance evaluations showed that the proposed system is able to produce a one-dimension (1D) chaotic system with better chaotic performances and larger chaotic ranges compared with the previous chaotic maps. Ref. [18] proposed a new chaotic system that utilizes two existing one-dimensional chaotic map levels combined into one, and although its complexity and sensitivity are improved compared to the original one-dimensional map, the size of the parameter space is still finite. Ref. [19] proposed a new two-dimensional sine improved logistic iterative chaotic map with infinite collapse (ICMIC) modulation map (2D-SLIM) based on the improved two-dimensional closed-loop modulation coupling model. The performance analysis results show that it has a large parameter space, large Lyapunov exponents, and high complexity. Ref. [20] proposed a sixth-order cellular neural network (CNN) hyperchaotic system, in addition, an octal number communication system based on this hyperchaotic system was given. This communication system has the features of large capacity of signal transmission, high security, and good expansibility. The original information signal can be covered and recovered effectively in this octal number communication system. Ref. [21] proposed a semi-symmetric image encryption scheme based on function projective synchronization between two hyperchaotic systems, and it has several advantages such as great speed and relatively low complexity compared respectively to symmetric and asymmetric algorithms. In particular, the key is generated simultaneously and independently in encryption and decryption sides, which effectively avoids the key transmission and threats of key exposure. Moreover, the sixth-order CNN is not only regarded as the drive system for key synchronization, but is also used for diffusing key generation to enhance the security and sensitivity of the scheme.

By comparing various encryption algorithms, Ref. [17] uses an improved chaotic system based on a one-dimensional chaotic system, which is a low-dimensional system with few control parameters, high real-time performance, and low computational cost, but the chaotic behavior with small key space needs to be improved. In Ref. [18], low-dimensional chaos is also used and has the same characteristics. In Refs. [12,19,20,21], high-dimensional chaos is used to compensate for the problems of low-dimensional chaotic key space and chaotic behavior. However, due to its complex structure and multiple parameters, it increases the difficulty and computational complexity of its hardware and software implementation, and its real-time performance is poor and time consumption is large. Therefore, it is necessary to use a chaotic system with few control parameters and complex chaotic behavior. In this paper, we propose a new chaotic system for a medical image encryption algorithm to address the above problems. The system consists of a one-dimensional chaotic map with one control parameter, and the bifurcation diagram, Lyapunov exponent, 0–1 test, and sample entropy analysis are performed to verify the good performance of the chaotic system. To compensate for the problem of small key space due to few control parameters, a key generation model is proposed, which not only keeps the advantages of simple and fast implementation of the original one-dimensional chaotic system, but also solves the shortcoming of insufficient key space of the one-dimensional chaotic system.

To boost encryption effectiveness, in addition to using chaotic systems with high real-time performance, the problem should be solved from the image itself, such as compressing and encrypting the image [22,23,24]. Unfortunately, most compression algorithms lead to lossy image decryption, and any degradation of image quality may lead to misdiagnosis, so most methods of image compression are not suitable for medical images. Image encryption algorithms are currently classified into two types: full image encryption, which encrypts the entire image, and selective image encryption, which encrypts only a portion of the image. Selective image encryption has been popular in recent years as a way to reduce image encryption and decryption processing time while maintaining a high level of security, and has received the attention of many scientists and engineers with several encryption schemes proposed [25,26,27]. For medical images, only part of an image is meaningful and the other parts are pure black regions, which can be divided into two parts, private and non-private regions, where the private region is also called the region of interest (ROI) and the non-private region is also called the region of background (ROB) [28]. The method for selecting the region of interest is just as crucial as the encryption algorithm in the region of interest encryption technique. ROI encryption techniques can be classified into two groups based on the privacy region selection method: manually selected encryption and automatically selected encryption. The manual selection of the ROI encryption approach entails the user determining a certain privacy zone and then encrypting that region [29,30]. This method is time-consuming and inaccurate. As a result, a plethora of automated selection schemes have been presented. A pixel-based scanning method is proposed in the literature [31] from the left and right sides to the center and a threshold value is used to determine the contour of the ROI. An algorithm for ROI determination of raw medical images based on block energy was proposed in the literature [32]. Refs. [33,34] set the size of ROI to a rectangle in medical images and then encrypts it, however, due to the shape and size of ROI, this solution is limited and cannot adequately secure personal information. Ref. [35] suggested a medical image encryption technique that is fully selective and chaotic. The approach is divided into rounds, and in the diffusion phase of each round, a pseudo-random matrix of the same size as the input image is used to improve the scheme’s speed while retaining good security. Ref. [36] proposed a game theory-based ROI optimized nondestructive medical image encryption and decryption scheme. The ROI parameters are optimized using a bargaining game. This allows accurate and adaptive determination of the region of interest, adapting to a wide range of medical image types and encryption requirements. However, this requires us to test all the pixel thresholds and finally select the most suitable segmentation threshold from all the results. To address the above, we design an efficient and secure image encryption algorithm using medical image characteristics. We combine image segmentation with image encryption by first segmenting the image adaptively, segmenting the ROI according to the obtained target segmentation threshold, and encrypting only the ROI, which reduces the processing delay and resource overhead.

Contribution:

(1) The 1D-$e^{\lambda }$-cos-cot (1D-ECC) chaotic map is proposed and the performance of the map is tested and analyzed, which has a wider range of control parameters. When compared to a traditional 1D chaotic system, this system has better real-time performance and chaotic behavior.
(2) A key generation model based on DICOM (Digital Imaging and Communications in Medicine) file information is proposed, and the initial values and control parameters of the 1D $e^{\lambda }$-cos-cot map (1D-ECC) chaotic map are generated using this model to further generate the keystream and expand the key space.
(3) A new 1D $e^{\lambda }$-cos-cot map (1D-ECC) medical image region of the interest-based encryption algorithm is proposed, and the simulation and security evaluation results show that the algorithm has high security and can resist common attacks.

The rest of this paper is organized as follows. The 1D $e^{\lambda }$-cos-cot (1D ECC) chaotic map will be introduced in Section 2 and its performance will be evaluated using a bifurcation diagram, Lyapunov exponent, 0–1 test, and sample entropy test. Section 3 will introduce the key generation model based on the DICOM file with a 1D-ECC chaotic map. Section 4 will cover the implementation of the entire encryption algorithm. Simulation results will be given in Section 5, and then the security performance of the 1D-ECC chaotic map based encryption algorithm will be further analyzed. Finally, the conclusion will be given in Section 7.

## 2. 1D-$e^{\lambda }$-cos-cot Map

In this section, we propose a new one-dimensional chaotic map to solve the problem of insufficient chaotic behavior of traditional one-dimensional chaotic maps and analyze its chaotic behavior.

### 2.1. Logistic Chaotic Map and Sine Map

**Figure 1:**
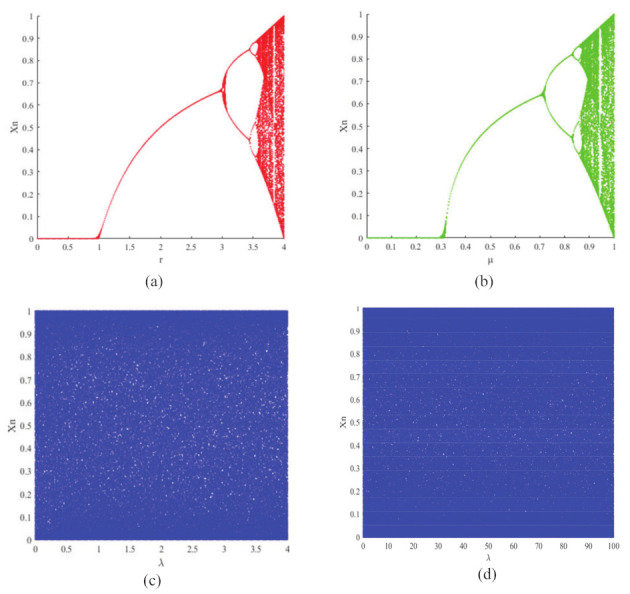
Bifurcation diagram of (**a**) logistic map with r∈(0,4); (**b**) sine map with μ∈[0,1]; (**c**) 1D-ECC map with λ∈[0,4]; and (**d**) 1D-ECC map with λ∈[0,100].

**Figure 2:**
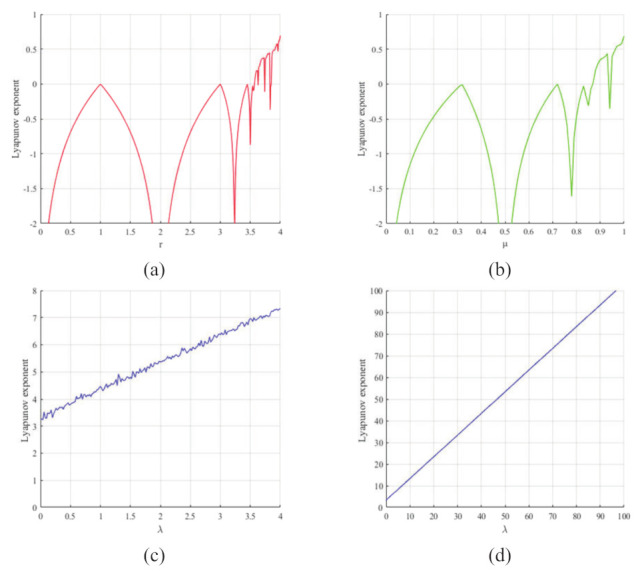
Lyapunov exponent of (**a**) logistic map with r∈(0,4); (**b**) sine map with μ∈[0,1]; (**c**) 1D-ECC map with λ∈[0,4]; and (**d**) 1D-ECC map with λ∈[0,100].

The logistic map and sine map are simple, nonlinear dynamical systems with complex chaotic behavior. They can be represented by the equation below [37]:

(1) $$x_{n+1}=r\left(1-x_{n}\right)x_{n}$$

(2) $$x_{n+1}=\mu \sin\left(\pi x_{n}\right)$$

where *r* is the control parameter in the range of r∈(0,4) and $x_{n}$ is the output chaotic sequence, whose initial value can be expressed by $x_{n=0}$. μ is the control parameter in the range μ∈[0,1] and $x_{n}$ is the output chaotic sequence, whose initial value can be initialized by $x_{n=0}$. Both the logistic map and sine map suffer from two security problems: first, they have a small effective range of control parameters, as shown in Figure 1a,b: only r∈[3.57,4] and μ∈[0.8722,1] show chaotic behavior, which leads to a small range of key values for cryptosystems that use the system as a random number generator. Further verification can be obtained from the Lyapunov exponential spectrum in Figure 2a,b. Secondly, the output chaotic sequences are unevenly distributed, with insufficient randomness and periodic problems, which will lead to security risks in the corresponding cryptosystems.

### 2.2. 1D-$e^{\lambda }$-cos-cot Map

The proposed new chaotic map is shown in Equation (3).

(3) $$x_{n+1}=\cos\left(e^{\lambda }\cot\left(2\pi x_{n}+1\right)\right)\;\mathrm{mod}\;1$$

where λ is the control parameter λ∈[0,+∞], *n* is the number of iterations, $x_{n}$ is the output chaotic sequence, $x_{n}\in \left[0,1\right]$, $x_{n+1}$ is the next state of the chaotic sequence, and mod() is the modulation function. In the next section, we will prove that the chaotic map has good chaotic performance when λ∈[0,+∞].

### 2.3. Performance Analysis

In this section, we carry out some performance analysis tests on the proposed new one-dimensional chaotic map, including the bifurcation diagram, Lyapunov exponent, 0–1 test, and sample entropy test, and compare the chaotic behavior with the traditional one-dimensional chaotic map.

#### 2.3.1. Bifurcation Diagram

As the parameters of a dynamical system change, the bifurcation event occurs. A chaotic system’s bifurcation diagram can be used to describe the resulting chaotic sequence and hence study the chaotic behavior of the system. Logistic map and sine map bifurcation diagrams are shown in Figure 1a,b. For the logistic map, a periodic window appears when r=3.5699456, and the system enters a chaotic state when r=3.57. For the sine chaos map, the system enters the chaotic state when μ=0.8722. It can be seen that these two chaotic maps exhibit chaotic performance only for a very small time interval. Moreover, the logistic chaos map and the sine chaos map are complete maps for the interval [0,1] only when r=4 and μ=1. On the contrary, the 1D-ECC map exhibits better chaotic properties. Figure 1c shows the chaotic behavior for λ∈[0,4]. From the comparison results we can observe that the 1D-ECC chaotic map has a greater range of the parameter; in comparison to the logistic and sine chaotic maps, the 1D-ECC chaotic map has a higher random-like performance; and unlike the logistic and sine maps, the 1D-ECC chaotic map has no period window. In addition, to better compare the effects of our proposed map, we also plot the bifurcation diagram for a larger range of control parameters, as shown in Figure 1d, and it can be seen that the 1D-ECC chaos map still has good chaotic properties when the control parameter λ∈[0,100].

#### 2.3.2. Lyapunov Exponent

In a dynamical system, the Lyapunov exponent can be used to measure the rate of separation between infinitely close trajectories. When the Lyapunov exponent is positive, the dynamical system can be considered to have chaotic behavior, and the larger the Lyapunov exponent the higher the chaotic complexity of the mixed system. We plot the Lyapunov exponents of three chaotic maps, and the Lyapunov exponent formula for a one-dimensional chaotic system is as follows [38]:

(4) $$\omega =\lim_{x\to \infty }\frac{1}{n}\sum_{i=0}^{n-1}\ln\left|f^{\prime }\left(x_{i}\right)\right|$$

As shown in Figure 2a, the Lyapunov exponents of the logistic map are less than zero when the parameter r<3.57 and, as shown in Figure 2b, the Lyapunov exponents of sine map are less than zero when the parameter μ<0.8722, which means they have no chaotic behavior. Meanwhile, in our proposed 1D-ECC map, as shown in Figure 2c, the Lyapunov exponents of the 1D-ECC map are all greater than zero when λ≥0, and the Lyapunov exponents increase gradually with the increase in the range of control parameters, as shown in Figure 2d; when λ=100, the Liapunov exponent exceeds 100. Compared with the logistic map and sine map, 1D-ECC has a greater parameter range and a higher Lyapunov exponent; it can be called a good chaotic map.

#### 2.3.3. 0–1 Test

The 0–1 test can be used to assess chaotic systems’ performance. The test uses chaotic systems to produce sequences for numerous rounds, and the test’s outcome, *K*, is either 0 or 1. For a real constant r∈(0,π), a number of iterations, *n*, and a chaotic sequence $x{\left(i\right)}_{i=1,2,3,\ldots ,n}$,*K* is calculated as follows [39]:

(5) $$Kc=log\frac{M_{\left(n\right)}}{logn}$$

Here

(6) $$M_{\left(n\right)}=\lim_{N\to \infty }\frac{1}{N}\sum_{i=1}^{n}{\left[p\left(i+n\right)-p\left(i\right)\right]}^{2}+{\left[q\left(i+n\right)-q\left(i\right)\right]}^{2}$$

and

(7) $$p_{\left(n\right)}=\sum_{i=1}^{n}X\left(i\right)cos\left(ir\right)$$

(8) $$q_{\left(n\right)}=\sum_{i=1}^{n}X\left(i\right)sin\left(ir\right)$$

**Figure 3:**
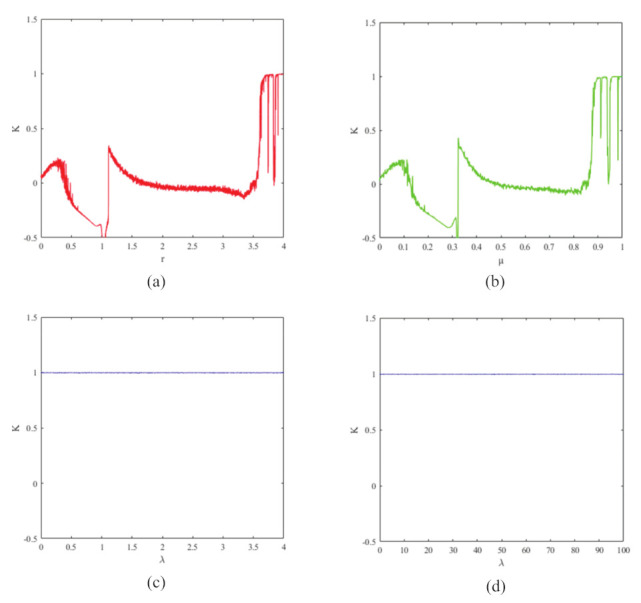
0–1 test of (**a**) logistic map with r∈(0,4); (**b**) sine map with μ∈[0,1]; (**c**) 1D-ECC map with λ∈[0,4]; and (**d**) 1D-ECC map with λ∈[0,100].

The system is chaotic when K≈1. In this paper, n=200, $r\in [\frac{\pi }{5},\frac{4\pi }{5}]$ is chosen to calculate the value of K for two different chaotic maps. The associated outcomes are given in Figure 3a which shows the results of a logistic map test, the value of *K* is close to 1 only when the parameter is larger than 3.6 and is unstable. For the sine map, as shown in Figure 3b, the *K* value is close to 1 only when its parameter is close to 1. In the test results of the 1D-ECC map shown in Figure 3c, the *K* value is always close to 1 when the parameter λ∈[0,100]. In Figure 3d, the *K* value is very close to 1 for λ∈[0,100], which indicates that the 1D-ECC map has better mixing performance than the existing logistic and sine map have better chaotic performance, so we can apply them to the generation of chaotic sequences.

#### 2.3.4. Sample Entropy

**Figure 4:**
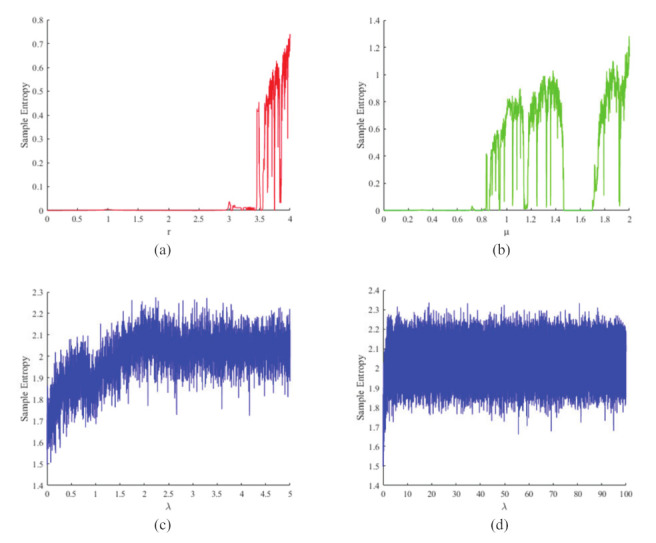
Sample entropy of (**a**) logistic map with r∈(0,4); (**b**) sine map with μ∈[0,1]; (**c**) 1D-ECC map with λ∈[0,4]; and (**d**) 1D-ECC map with λ∈[0,100].

Sample entropy is a type of entropy used to assess the complexity of a time series [40,41,42]. In the field of chaos, the sample entropy is usually used to assess the complexity of the output signal of a dynamic system. When the SE is large, it indicates that the signal is less regular, and the chaotic system has more complex behavior. For the logistic map, when r>3.57, the sample entropy value starts to increase, and when r=4, the sample entropy value is about 0.8, as shown in Figure 4a. For the sine map, the sample entropy value starts to increase when μ>0.872, and when r=1, the sample entropy value is about 0.8, as shown in Figure 4b. While our proposed 1D-ECC map, the sample entropy value reaches about 1.8 at λ=0 and increases with the increase in the control parameter λ, as shown in Figure 4c. In addition, we plotted the sample entropy for a larger range of control parameters, and the sample entropy value remains around 2 when λ=100, as shown in Figure 4d. It can be seen that the 1D-ECC map achieves positive sample entropy values for all control parameters and has larger sample entropy values than the logistic and sine map, indicating that the 1D-ECC map has better chaotic performance.

## 3. DICOM File-Based Key Generation Model with 1D-ECC Chaotic Map

In this section, the DICOM standard is introduced and 1D-ECC is applied to generate encryption keys based on DICOM data information.

### 3.1. Introduction of DICOM

**Figure 5:**
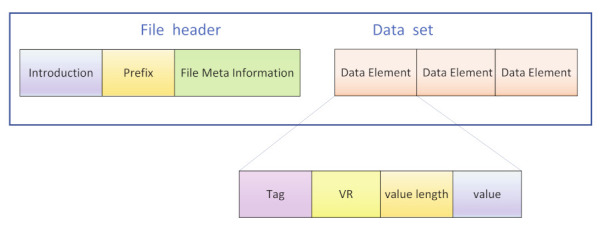
Structure of DICOM file.

The DICOM (Digital Imaging and Communications in Medicine) standard is a standard for the communication and administration of medical imaging information and related data [43]. The overall structure of a DICOM file is shown in Figure 5. Each DICOM file must include a header, and the DICOM file header contains relevant information that identifies the data set. The main feature of DICOM format medical image files is that they contain not only basic information such as image size, width, height, and number of bits per pixel, but also a lot of medical information in the data elements of the data set, such as hospital name, test type, basic patient information, doctor information, etc.

The main component of a DICOM file is the data set, which consists of data elements arranged in a prescribed order, the most basic unit being the data element. For DICOM files, which are usually transferred explicitly, the data elements are arranged in the order of the tags from smallest to largest. It consists of four main parts:

(1) DICOM Tag: the identification of the information stored.
(2) VR (value representation): store the data type describing the information.
(3) Value length: store the length of the data describing the information.
(4) Value: store the data value describing the information.

**Figure 6:**
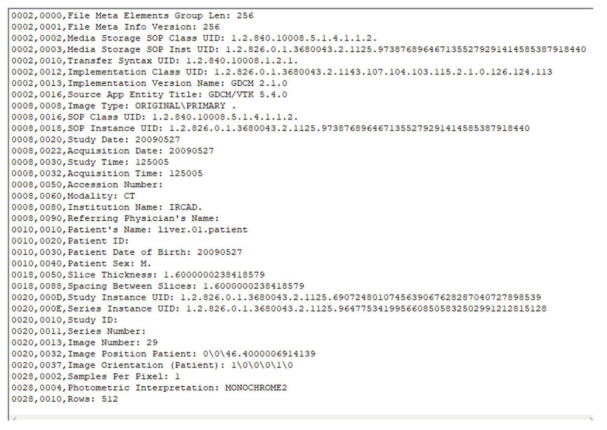
Information data elements of DICOM file.

DICOM Tags are classified into four groups: (a) Patient, (b) Study, (c) Series, and (d) Image’s Name. Figure 6 shows some of the data element information in the DICOM image. As shown in Figure 6, each DICOM Tag is identified by the combination of two hexadecimal numbers, Group and Element. For example, the tag (0010,0010) represents the Patient’s Name, which stores the Patient Name of this DICOM image, and the tag (0010, 0030) represents the Patient Date of Birth, which stores the date of birth of the patient for this DICOM image. In DICOM, the data values stored in each tag are different. Therefore, we mainly apply the data value stored in the data element describing this information and use the data value as the key to 1D-ECC chaos mapping after processing, which ensures that the plaintext ciphertext is highly correlated and further improves the security of the algorithm.

### 3.2. Key Generation

As the encrypted image is closely related to the key, it is very important to use a good key generation algorithm. First, set the control parameter $\lambda_{0}$ and the initial value $x_{0}$ for the 1D-ECC chaotic map as the user key. To generate the random number sequence A, the chaotic map is iterated M×N times. To avoid transient effects, the first 1000 sequences of random number sequence A are discarded to obtain the random number sequence B. The first six elements of the random number sequence B are selected as $b_{1},b_{2},b_{3},b_{4},b_{5},b_{6}$, which are used to control the selection of DICOM tag information. For $b_{1},b_{2},b_{3},b_{4},b_{5},b_{6}$, the following operations are performed to obtain $b_{1}^{\prime },b_{2}^{\prime },b_{3}^{\prime },b_{4}^{\prime },b_{5}^{\prime },b_{6}^{\prime }$.

(9) $$\left\{\begin{array}{c} b_{1}^{\prime }=\;\mathrm{mod}\;\left(\mathrm{floor}\;\left(b_{1}\times 10^{9}\right),S\right)\\ b_{2}^{\prime }=\;\mathrm{mod}\;\left(\mathrm{floor}\;\left(b_{2}\times 10^{9}\right),S\right)\\ b_{3}^{\prime }=\;\mathrm{mod}\;\left(\mathrm{floor}\;\left(b_{3}\times 10^{9}\right),S\right)\\ b_{4}^{\prime }=\;\mathrm{mod}\;\left(\mathrm{floor}\;\left(b_{4}\times 10^{9}\right),S\right)\\ b_{5}^{\prime }=\;\mathrm{mod}\;\left(\mathrm{floor}\;\left(b_{5}\times 10^{9}\right),S\right)\\ b_{6}^{\prime }=\;\mathrm{mod}\;\left(\mathrm{floor}\;\left(b_{6}\times 10^{9}\right),S\right) \end{array}\right.$$

where *S* is the total number of DICOM data elements, $b_{1}^{\prime },b_{2}^{\prime },b_{3}^{\prime },b_{4}^{\prime },b_{5}^{\prime },b_{6}^{\prime }$, as a control pointer for DICOM data element selection, and six sets of DICOM data element information can be obtained. For example, the information obtained is:

(1) (0008,0018): SOP Instance UID (Instance UID number); UID form is a string used to uniquely identify various different information objects in the DICOM standard, such as the value representation type of data elements, DICOM abstract syntax name, transmission syntax, application context name to ensure uniqueness in various different countries, regions, manufacturers, and equipment use.
(2) (0010,0010): Patient’s Name.
(3) (0010,0030): Patient Date of Birth (the patient’s date of birth).
(4) (0010,0020): Patient ID (patient’s ID).
(5) (0008,0023): Image Date (image date).
(6) (0008,0050): Accession Number (registration number).

**Figure 7:**
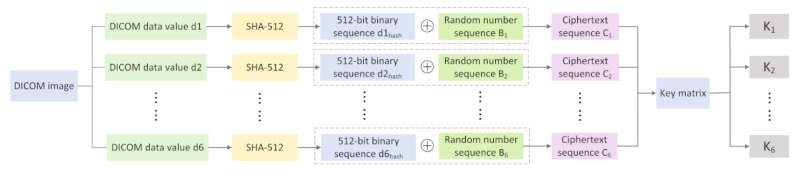
Key generation scheme.

As the information in the DICOM image is public, to improve the security of the key, we carry out XOR encryption on the selected data value and use the encrypted DICOM data value to generate the key used in the subsequent encryption process, as shown in Figure 7 The specific operations are as follows:

Step 1: The selected six groups of DICOM Tag information are noted as d1,d2,d3,d4,d5,d6;

Step 2: The hash function is irreversible, conflict-resistant, covert, and can effectively withstand known plaintext attacks and selected plaintext/ciphertext attacks when compared to the random key generation approach. Therefore, we operate on the data using SHA-512, which is part of a set of hashing algorithms called SHA-2 that performs a hash function on the given data and yields a hexadecimal string of 128 bits in length. Using this to operate on d1,d2,d3,d4,d5,d6, a hexadecimal hash string of 128 bits in length is obtained and the hash value is noted as $d1_{\mathrm{hash}}$, $d2_{\mathrm{hash}}$, $d3_{\mathrm{hash}}$, $d4_{\mathrm{hash}}$, $d5_{\mathrm{hash}}$, and $d6_{\mathrm{hash}}$, respectively, as shown in the following equation:

(10) $$\left\{\begin{array}{c} d1_{\mathrm{hash}}=\;\mathrm{SHA}\text{-}\;512\left(d1\right)\\ d2_{\mathrm{hash}}=\;\mathrm{SHA}\text{-}\;512\left(d2\right)\\ d3_{\mathrm{hash}}=\;\mathrm{SHA}\text{-}\;512\left(d3\right)\\ d4_{\mathrm{hash}}=\;\mathrm{SHA}\text{-}\;512\left(d4\right)\\ d5_{\mathrm{hash}}=\;\mathrm{SHA}-\;512\left(d5\right)\\ d6_{\mathrm{hash}}=\;\mathrm{SHA}\text{-}\;512\left(d6\right) \end{array}\right.$$

Step 3: Convert $d1_{\mathrm{hash}}$, $d2_{\mathrm{hash}}$, $d3_{\mathrm{hash}}$, $d4_{\mathrm{hash}}$, $d5_{\mathrm{hash}}$, and $d6_{\mathrm{hash}}$ from hexadecimal to binary, as one hexadecimal number is equal to four binary numbers, yielding six binary sequences of length 512;

Step 4: Convert the random number sequence B from decimal to binary; for the random number B, intercept every 512 bits, intercept six times, and obtain six binary chaotic sequences of length 512 as $B_{1},B_{2},B_{3},B_{4},B_{5},B_{6}$;

Step 5: $d1_{\mathrm{hash}}$, $d2_{\mathrm{hash}}$, $d3_{\mathrm{hash}}$, $d4_{\mathrm{hash}}$, $d5_{\mathrm{hash}}$, and $d6_{\mathrm{hash}}$ and $B_{1},B_{2},B_{3},B_{4},B_{5},B_{6}$ are subjected to heteroskedastic operation (⊕) to obtain six ciphertext sequences $C_{1},C_{2},C_{3},C_{4},C_{5},C_{6}$;

(11) $$\left\{\begin{array}{c} C_{1}=d1_{\mathrm{hash}}⨁B_{1}\\ C_{2}=d2_{\mathrm{hash}}⨁B_{2}\\ C_{3}=d3_{\mathrm{hash}}⨁B_{3}\\ C_{4}=d4_{\mathrm{hash}}⨁B_{4}\\ C_{5}=d5_{\mathrm{hash}}⨁B_{5}\\ C_{6}=d6_{\mathrm{hash}}⨁B_{6} \end{array}\right.$$

Step 6: divide $C_{1},C_{2},C_{3},C_{4},C_{5},C_{6}$, i.e., each ciphertext sequence, into 32 groups (the length of each group is 16 bits), and convert them from binary to decimal to find a 6×32 decimal matrix key;

(12) $$key=\left[\begin{array}{cccc} c_{1,1} & c_{1,2} & \cdots \cdots & c_{1,32}\\ c_{2,1} & c_{2,2} & \cdots \cdots & c_{2,32}\\ c_{3,1} & c_{3,2} & \cdots \cdots & c_{3,32}\\ c_{4,1} & c_{4,2} & \cdots \cdots & c_{4,32}\\ c_{5,1} & c_{5,2} & \cdots \cdots & c_{5,32}\\ c_{6,1} & c_{6,2} & \cdots \cdots & c_{6,32} \end{array}\right]$$

Step 7: Perform a dissimilarity operation on each element of the key in each row;

(13) $$\left\{\begin{array}{c} k_{1}=c_{1,1}⊕c_{1,2}⊕\cdots \cdots ⊕c_{1,32}\\ k_{2}=c_{2,1}⊕c_{2,2}⊕\cdots \cdots ⊕c_{2,32}\\ k_{3}=c_{3,1}⊕c_{3,2}⊕\cdots \cdots ⊕c_{3,32}\\ k_{4}=c_{4,1}⊕c_{4,2}⊕\cdots \cdots ⊕c_{4,32}\\ k_{5}=c_{5,1}⊕c_{5,2}⊕\cdots \cdots ⊕c_{5,32}\\ k_{6}=c_{6,1}⊕c_{6,2}⊕\cdots \cdots ⊕c_{6,32} \end{array}\right.$$

Step 8: According to the obtained $k_{1},k_{2},k_{3},k_{4},k_{5},k_{6}$, to carry out the operation of Formula (13) to obtain the subsequent encryption process requires the control parameters $\lambda_{0},\lambda_{1},\lambda_{2}$ and the initial value initial value $x_{0},x_{1},x_{2}$ specific operation, as shown in (14).

(14) $$\left\{\begin{array}{c} \lambda_{0}=\left(\frac{\mathrm{sum}(\mathrm{key}(1:))}{k_{1}+k_{2}+k_{3}}\right)\\ \lambda_{1}=\left(\frac{\mathrm{sum}(key(2:))}{k_{2}+k_{3}+k_{4}}\right)\\ \lambda_{2}=\left(\frac{\mathrm{sum}(\mathrm{key}(3:))}{k_{3}+k_{4}+k_{5}}\right)\\ x_{0}=\left(\frac{\max((\mathrm{key}(4:)))+\min((key(4:)))}{k_{1}+k_{2}+k_{3}+k_{4}}+\left(e-\mathrm{floor}\;\left(e\right)\right)\right)\;\mathrm{mod}\;1\\ x_{1}=\left(\frac{\max((key(5:)))+\min((key(5:)))}{k_{2}+k_{3}+k_{4}+k_{5}}+\left(e-\mathrm{floor}\;\left(e\right)\right)\right)\;\mathrm{mod}\;1\\ x_{2}=\left(\frac{\max((key(6:)))+\min((key(6:)))}{k_{3}+k_{4}+k_{5}+k_{6}}+\left(e-\mathrm{floor}\;\left(e\right)\right)\right)\;\mathrm{mod}\;1 \end{array}\right.$$

where *e* is the information entropy of the region of interest in the image to be encrypted, floor() is the downward rounding function, and mod() is the modulo function. The addition of e ensures that the key of each encryption is highly dependent on the plaintext image and improves the correlation between the ciphertext and the plaintext.

### 3.3. Generation of Chaotic Sequences

Using the steps outlined above, the initial values of control parameters $\lambda_{0},\lambda_{1},\lambda_{2}$ and the initial values $x_{0},x_{1},x_{2}$ are obtained, and the chaotic sequences are generated as follows:

Step 1: Substitute the obtained parameters $\lambda_{0}$ and the initial value $x_{0}$ into the system and iterate M×N times to obtain the chaotic sequence $A_{1}$;

Step 2: Substitute the obtained parameter $\lambda_{1}$ and the initial value $x_{1}$ into the system, and iterate 2×M×N times to obtain the chaotic sequence $A_{2}$;

Step 3: Substitute the obtained parameter $\lambda_{2}$ and the initial value $x_{2}$ into the system, and iterate 2×M×N times to obtain the chaotic sequence $A_{3}$;

Step 4: Arrange the chaotic sequence $A_{1}$ in ascending order and record its index sequence T1;

Step 5: The chaotic sequences $A_{2}$ and $A_{3}$ are operated as shown in (15) and (16) to obtain the diffusion key stream $Q_{1}$ and $Q_{2}$, respectively;

(15) $$\begin{array}{cc} \; & Q_{1}=int16\left(mod\left(floor\left(A_{3}\times 10_{.}^{9}\right),65536\right)\right. \end{array}$$

(16) $$\begin{array}{cc} \; & Q_{2}=int16\left(mod\left(floor\left(A_{4}\times 10_{.}^{9}\right),65536\right)\right. \end{array}$$

Step 6: Divide the chaotic sequence $Q_{1}$ into two chaotic sequences $Q_{11},Q_{12}$ of length M×N;

Step 7: Divide the chaotic sequence $Q_{2}$ into two chaotic sequences $Q_{21},Q_{22}$ of length M×N;

Among them, the index sequence $T_{1}$ is used for the first permutation, the chaotic sequences $Q_{11},Q_{12},Q_{21},Q_{22}$ are used for the two-dimensional bidirectional diffusion.

## 4. Selection of Region of Interest Blocks

In this section, the selection of target thresholds and the process of determining the region of interest (ROI) are mainly described. The OTSU threshold segmentation method is used in the region of interest block selection process to segment the region of interest in the picture and acquire the target threshold t, which will be used for the later judgment of the region of interest.

### 4.1. Target Threshold Segmentation

The OTSU algorithm is a fast approach for binarizing images that was proposed by Japanese researcher Otsu in 1979 [44], and it is based on the clustering concept:

(1) Dividing the number of gray levels of an image into two parts by gray level, such that the difference in gray values between two parts is the largest and the difference in gray levels between each part is the smallest.
(2) Finding a suitable gray level to divide by the calculation of a variance.
(3) The traversal method is used to obtain the threshold t that maximizes the variance between classes, and t is the desired target threshold.

The OTSU method is often regarded as the best technique for image segmentation threshold selection because it is simple to implement and is unaffected by picture brightness or contrast, making it ideal for ROI segmentation in medical images.

### 4.2. Selection of Region of Interest

Step 1: The size of the plaintext image is M×N, and the plaintext image is partitioned into blocks of size n×n (*n* is an integer that can be divided by *M* and *N*), which are divided into *S* blocks, $S=\frac{M\times N}{n^{2}}$. Here, we set *n* to 4, and the size of each image block is 4×4=16.

Step 2: calculate the average of grayscale pixels for each image block according to Equation (17).

(17) $${\bar{v}}_{s}=mean\left(B_{i}\right)$$

where mean() is the mean function and $B_{i}$ denotes the *i*-th image block, i=1,2,3,…,S.

Step 3: The identification method of the region of interest is as follows:

When ${\bar{v}}_{s}>t$, then the image block $B_{s}$ is the region of interest $ROIB_{r}$, and the corresponding region of interest flag bit $ROI_{flag_{s}}$ can be set to 1.

When ${\bar{v}}_{s}\leq t$, the image block $B_{s}$ is a non-interest region RONI, and the corresponding region of interest flag bit $ROI_{flag_{s}}$ can be set to 0.

Where t is the threshold value for determining the ROI region, which is obtained by thresholding the image by the above OTSU threshold segmentation method; where *r* is the image region of interest number, $r=1,2,\cdots ,\mathrm{sum}\left(ROI_{flag_{s}}\right)$; where $ROI_{flag_{s}}$ is the corresponding flag bit of the s region of interest; and where $\mathrm{sum}\left(ROI_{flag_{s}}\right)$ is the number of all image blocks in the image that are discriminated as regions of interest.

**Figure 8:**
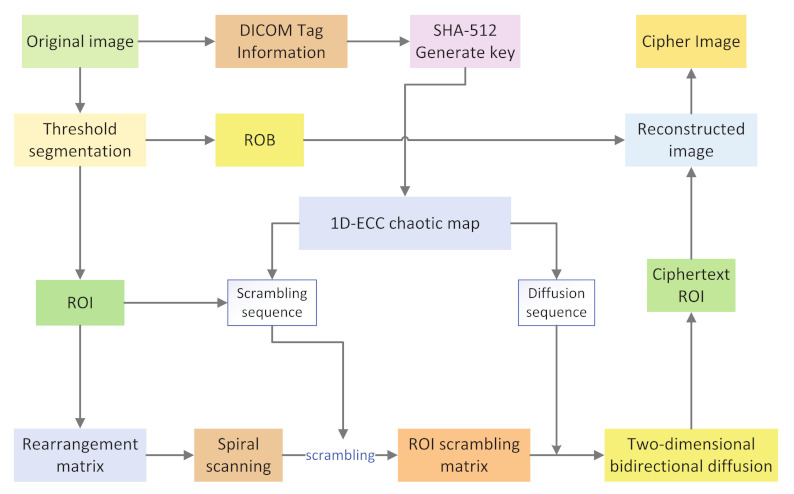
Encryption flow chart.

Step 4: The r region of interest sequence $ROIB_{r}$ are combined into a matrix in a top-down order to obtain the region of interest matrix $ROI_{M}$ with length (n×n) and width $Col_{ROI}$, operating as shown in Figure 8.

(18) $$ROI_{M}=\left[ROIB_{1},ROIB_{2},\cdots ,ROIB_{\left(n\times n\right)\times Col_{ROI}}\right]$$

## 5. Region of Interest Encryption and Decryption Algorithm

### 5.1. Encryption Algorithm

The matrix $ROI_{M}$ obtained in Section 4.2 is converted into a signed hexadecimal integer, and then chaotic encryption is carried out. In image encryption, two very important operations are scrambling and diffusion. The goal of scrambling is to disrupt the relationship between image pixel positions. Diffusion operation ensures that even small changes in the original image can cause significant changes in the ciphertext image. We use helical scanning scrambling and two-dimensional bidirectional diffusion to encrypt the region of interest in the encryption stage.

#### 5.1.1. Scrambling Stage

**Figure 9:**
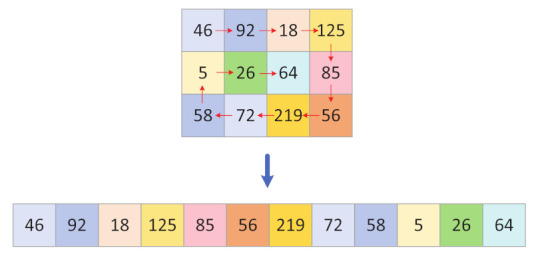
Spiral scan.

Step 1: The obtained region of interest matrix ROI is spirally scanned, and the scanning process is shown in Figure 9.

Step 2: The sequence of elements after scanning is output as a one-dimensional matrix sequence $ROI_{1D}$.

Step 3: The chaotic sequence $A_{1}$ is sorted in ascending order to obtain the index sequence index.

**Figure 10:**
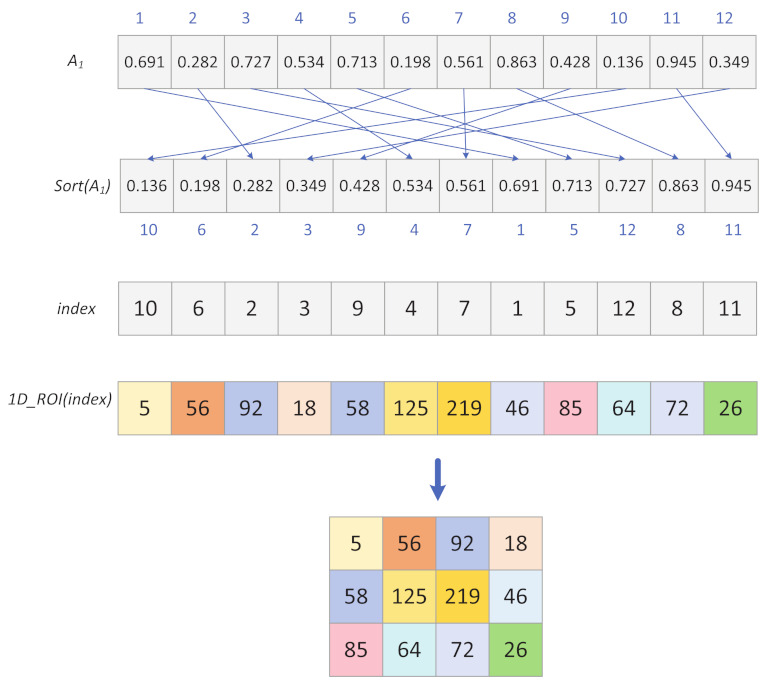
Scrambling process.

Step 4: Scramble $ROI_{1D}$ using an index sequence, to find the disordered one-dimensional matrix sequence $SROI_{1D}$ and the process is shown in Figure 10.

(19) $$SROI_{1D}=ROI_{1D}\left(index\right)$$

Step 5: $SROI_{1D}$ is deformed into a matrix $L_{R}OI$ of size $\left(n\times n\right)\times Col_{ROI}$.

(20) $$L_{ROI}=reshape\left(SROI_{1D},n\times n,Col_{ROI}\right)$$

#### 5.1.2. Diffusion Stage

**Figure 11:**
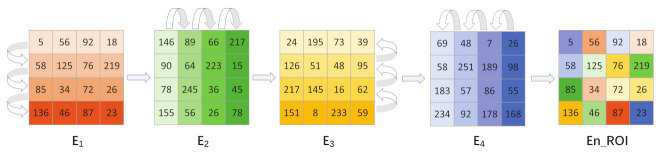
Diffusion process.

In the diffusion stage, two-dimensional bidirectional mode taking diffusion is adopted, that is, two-way column and column plus mode diffusion is carried out on the scrambled matrix. First, the scrambled matrix is spread row by row from top to bottom, that is, the first and second rows are added mode diffusion, and the result and the third row are added mode diffusion, until the diffusion of the last row is completed. Then, the same method spreads from left to right, column by column, until it ends in the rightmost column. We continue to add mode diffusion row by row from bottom to top until the diffusion of the first row is completed. Finally, we encrypt diffusion column by column from right to left until the diffusion of the left-most column is completed, and we finally obtain the ciphertext ROI matrix. The diffusion process is shown in Figure 11 and the specific steps are as follows:

Step 1: The chaotic sequences $Q_{11},Q_{12},Q_{21},Q_{22}$ are intercepted to obtain four chaotic sequences $K_{1},K_{2},K_{3},K_{4}$ of length $\left(n\times n\right)\times Col_{ROI}$, respectively;

Step 2: The chaotic sequences $K_{1},K_{2},K_{3},K_{4}$ are deformed to find the diffusion key matrix P1,P2,P3,P4 of size $\left(n\times n\right)\times Col_{ROI}$, respectively;

(21) $$P1=reshape(K_{1},n\times n,Col_{ROI})$$

(22) $$P2=reshape(K_{2},n\times n,Col_{ROI})$$

(23) $$P3=reshape(K_{3},n\times n,Col_{ROI})$$

(24) $$P4=reshape(K_{4},n\times n,Col_{ROI})$$

Step 3: The scrambled matrix sequence $L_{R}OI$ and diffusion key matrix P1 are carried out from top to bottom; mode-taking row diffusion and the matrix $E_{1}$ after the first diffusion is obtained:

(25) $$\begin{array}{cc} \; & E_{1}\left(1,:\right)=\;\mathrm{mod}\;\left(12+P1\left(1,:\right)+L_{-}ROI\left(1,:\right),65536\right)\\ \; & E_{1}\left(i,:\right)=\;\mathrm{mod}\;\left(E_{1}\left(i-1,:\right)+P1\left(i,:\right)+L_{-}ROI\left(i,:\right),\right.\\ \; & \left.65536\right) \end{array}$$

where i=2,3,⋯⋯n×n;

Step 4: Diffuse the matrix $E_{1}$ with the diffusion key matrix P2 from left to right to obtain the matrix $E_{2}$ after the second diffusion:

(26) $$\begin{array}{cc} \; & E_{2}(\left(:,j\right)=\;\mathrm{mod}\;\left(122+P2\left(:,1\right)+E_{1}\left(:,1\right),65536\right)\\ \; & E_{2}(\left(:,j\right)=\;\mathrm{mod}\;\left(E_{2}\left(:,j-1\right)+P2\left(:,j\right)+E_{1}\left(:,j\right),\right.\\ \; & \left.65536\right) \end{array}$$

where $j=2,3,\cdots \cdots Col_{ROI}$;

Step 5: Take the mode line diffusion of matrix $E_{2}$ and diffusion key matrix P3 from bottom to top and find the matrix $E_{3}$ after the third diffusion:

(27) $$\begin{array}{cc} \; & E_{3}\left(n\times n,:\right)=\;\mathrm{mod}\;\left(107+P3\left(n\times n,:\right)+E_{2}\left(n\times n,:\right),\right.\\ \; & \left.65536\right)\\ \; & E_{3}\left(i,:\right)=\;\mathrm{mod}\;\left(E_{3}\left(i+1,:\right)+P3\left(i,:\right)E_{2}\left(i,:\right),65536\right) \end{array}$$

where i=n×n−1,⋯⋯2,1;

Step 6: Diffuse the matrix $E_{3}$ with the diffusion key matrix P4 by taking the modulo columns from right to left to find the final ciphertext matrix $E_{4}$:

(28) $$\begin{array}{cc} \; & E_{4}(\left(:,Col_{ROI}\right)=\;\mathrm{mod}\;\left(208+P4\left(:,Col_{ROI}\right)+E_{3}\left(:,Col_{ROI}\right)\right.,\\ \; & \left.65536\right)\\ \; & E_{4}(\left(:,j\right)=\;\mathrm{mod}\;\left(E_{4}\left(:,j+1\right)+P4\left(:,j\right)+E_{3}\left(:,j\right),65536\right) \end{array}$$

where $j=Col_{ROI}-1\cdots \cdots 2,1$;

Step 7: $E_{4}$ is the final encrypted 16-bit unsigned integer region of interest ciphertext $En_{ROI}$, and the final encrypted image. EnImage is obtained by putting back the position of the element whose region of interest flag bit $ROI_{flag_{s}}$ is 1.

### 5.2. Decryption Algorithm

**Figure 12:**
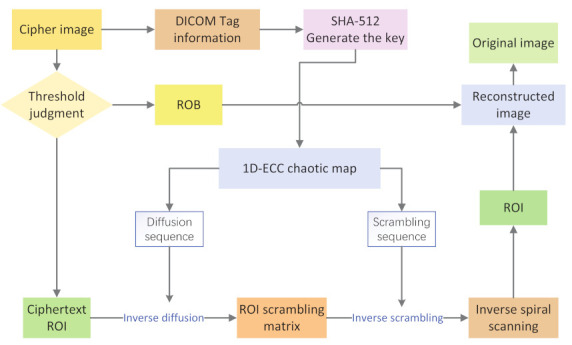
Decryption flow chart.

As the encryption/decryption scheme proposed in this paper is symmetric, the decryption process is the inverse process of the encryption process. The decryption flow chart is shown in Figure 12. As we convert the region of interest from 8 bits to 16 bits during encryption, the pixel value range of the ciphertext ROI is [0, 65,535], and the pixel value range of ROB is [0, 255], we can still use the block-based selection scheme to distinguish the encrypted ROI and ROB, and we can avoid passing the location information of ROI to the receiver or embedding the location information of ROI in the image. This approach reduces the possibility of leaking the encrypted location information during transmission and storage.

## 6. Simulation Results and Safety Analysis

**Figure 13:**
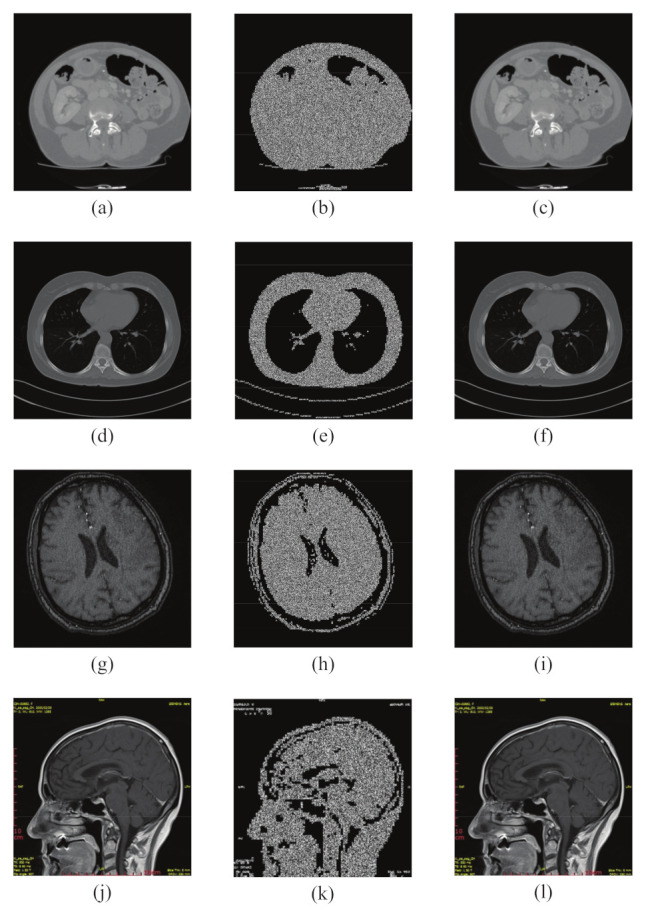
Encryption/decryption results: (**a**,**d**,**g**,**j**) show the plain images of four different structures, (**a**) CT Abdomen, (**d**) CT Lung, (**g**) CT Brain, and (**j**) MRI Brain. (**b**,**e**,**h**,**k**) are the corresponding ciphertext images. (**c**,**f**,**i**,**l**) are the decrypted images.

To verify the excellent performance of our proposed encryption scheme, a series of experiments are carried out in this section using MATLAB R2015 software, as shown in Figure 13. In addition, although the experiments in this section are analyzed only for grayscale medical images, our encryption algorithm is also applicable to RGB color medical images by using this encryption algorithm only for their three color components.

### 6.1. Simulation Results

To verify the excellent performance of our proposed encryption scheme, a series of experiments are conducted in this section using MATLAB R2015b software, and we use medical images from different parts of the body for testing, including abdominal data from MICCAI 2017 Liver Tumor Segmentation Challenge (LiTS) dataset, lung data from the Lung Image Database Consortium (LIDC-IDRI), and brain data from the Second Hospital of Jilin University. Although the experiments in this section are only conducted with grayscale medical images as an example for analysis, our encryption algorithm is also applicable to RGB color medical images by using this encryption algorithm for only their three color components. We used CT Abdominal, CT Lung, and CT Brain with a size of 512 × 512, and MRI Brain with a size of 320 × 320 for MATLAB simulation test, and obtained the region of interest segmentation threshold values of 66, 53, 45, and 55, respectively, as shown in Figure 13.

### 6.2. Key Space Analysis

The key space is the range of encryption key sizes and consists of all available keys used in the encryption process. In general, a bigger key space can withstand more exhaustive attacks, improving the algorithm’s security. When the key space of the algorithm is larger than $2^{100}$ [45] it can withstand brute force attacks. The encryption algorithm we suggest is as follows. There are eight keys: $\lambda_{0},\lambda_{1},\lambda_{2},\lambda_{3},x_{0},x_{1},x_{2},x_{3}$ assuming the computational accuracy of double-precision numbers is $10^{-15}$, so the total key space is ${\left(10^{15}\right)}^{8}=2^{398}>2^{100}$. As a result, the suggested picture algorithm has a wider key space, which makes it more resistant to brute force attacks.

### 6.3. Key Sensitivity Analysis

A good cryptosystem needs to not only be large enough, but also to be sensitive enough to the key. Key sensitivity means that even a very small change in the key bit can have a large impact on the entire cryptographic image, resulting in a completely different encrypted image [46]. We analyze the effect of key changes on the outcomes of both the encryption and decryption processes. Different encryption keys will result in different encryption results from the standpoint of the encryption process; from the standpoint of the decryption process, if the decryption key is different from the encryption key, it should not be possible to decrypt the original image correctly. Given $KEY=\lambda_{0},\lambda_{1},\lambda_{2},\lambda_{3},x_{0},x_{1},x_{2},x_{3}$ = 5.2, 15.19, 13.98, 11.65, 0.18, 0.35, 0.31, 0.38, for which six parameters are finely changed to obtain six different sets of keys, K1,K2,K3,K4,K5,K6, these six sets of keys are tested for key sensitivity.

$$\left\{\begin{array}{c} K1=\left\{\lambda_{0},\lambda_{1}+10^{-15},\lambda_{2},\lambda_{3},x_{0},x_{1},x_{2},x_{3}\right\}\\ K2=\left\{\lambda_{0},\lambda_{1},\lambda_{2}+10^{-15},\lambda_{3},x_{0},x_{1},x_{2},x_{3}\right\}\\ K3=\left\{\lambda_{0},\lambda_{1},\lambda_{2},\lambda_{3}+10^{-15},x_{0},x_{1},x_{2},x_{3}\right\}\\ K4=\left\{\lambda_{0},\lambda_{1},\lambda_{2},\lambda_{3},x_{0},x_{1}+10^{-15},x_{2},x_{3}\right\}\\ K5=\left\{\lambda_{0},\lambda_{1},\lambda_{2},\lambda_{3},x_{0},x_{1},x_{2}+10^{-15},x_{3}\right\}\\ K6=\left\{\lambda_{0},\lambda_{1},\lambda_{2},\lambda_{3},x_{0},x_{1},x_{2},x_{3}+10^{-15}\right\} \end{array}\right.$$

**Figure 14:**
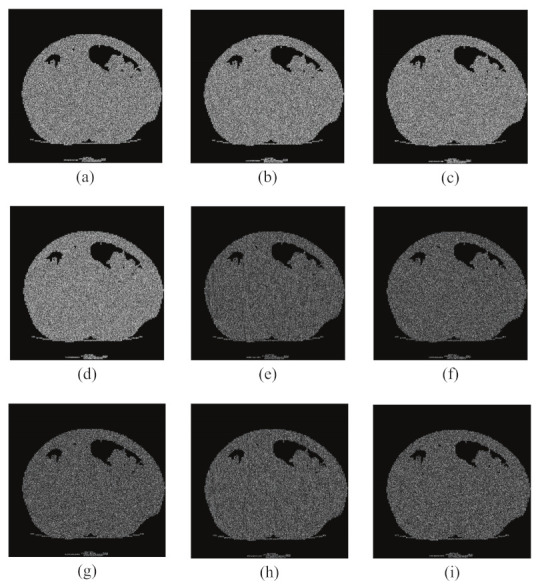
Key sensitivity test for CT Abdomen in encryption processes. (**a**) Encrypted image with KEY; (**b**) Encrypted image with KEY1; (**c**) Encrypted image with key KEY2; (**d**) Encrypted image with key KEY3; (**e**) difference between (**a**) and (**b**); (**f**) difference between (**a**) and (**c**); (**g**) difference between (**b**) and (**c**); (**h**) difference between (**b**) and (**d**); and (**i**) difference between (**c**) and (**d**).

**Figure 15:**
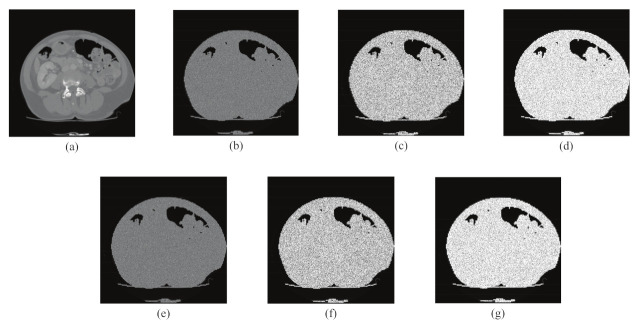
Key sensitivity test for CT Abdomen in decryption processes. (**a**) Decrypted image with correct KEY; (**b**) decrypted image with key KEY1; (**c**) decrypted image with KEY2; (**d**) decrypted image with key KEY3; (**e**) decrypted image with key KEY4; (**f**) decrypted image with key KEY5; and (**g**) decrypted image with key KEY6.

In the encryption process, we encrypted the same plaintext image, “CT Abdomen”, with a precision difference of $10^{-15}$, and then compared the encrypted images by subtraction, and the results are shown in Figure 14. The NPCR and UACI values between the ciphertext image generated with the correct key and the ciphertext image generated with the error key were calculated, and the results are listed in Table 1. In the decryption process, we use a key with an accuracy difference of $10^{-15}$, and the experimental results are shown in Figure 15, The results show that only the correct key can recover, and even if the key differs from the correct key by only $10^{-15}$, it is impossible to obtain the correct decryption result. These two experiments further demonstrate that the algorithm is highly sensitive to keys and can effectively resist brute force attacks.

### 6.4. Histogram Analysis

The histogram depicts the distribution of image pixels, which is a key indicator of the encryption algorithm’s performance [47]. The distribution of pixels in the original image is not uniform, in general. The pixels in the ciphertext should be dispersed as equally as possible to make the encrypted image more resistant to statistical analysis attacks. As a result, the better the encryption algorithm performs, the more uniform the histogram distribution of the ciphertext is. The histogram distribution of the ordinary image is uneven, whereas the histogram distribution of the encrypted image is uniform, indicating that statistical analysis is difficult for an attacker to acquire useful information.

**Figure 16:**
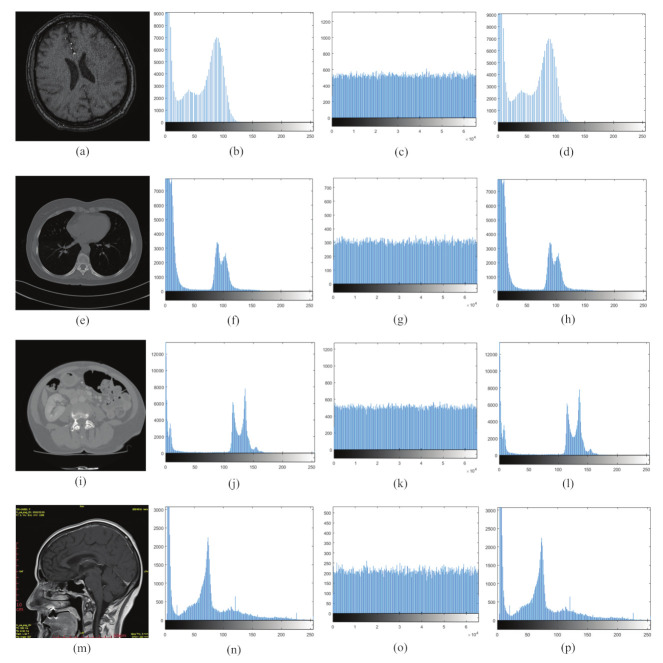
Histogram analysis of encryption/decryption results: (**a**,**e**,**i**,**m**) plaintext image; (**b**,**f**,**j**,**n**) histogram of plaintext image; (**c**,**g**,**k**,**o**) histogram of ciphertext image; and (**d**,**h**,**l**,**p**) histogram of decrypted image.

It can be seen that the histogram is more stable when the number of pixels per gray level is closer. It indicates that the value of each pixel tends to be consistent and the security is high. The results of histogram analysis are shown in Figure 16. It can be seen that, after encryption, the original uneven pixel distribution becomes smoother and the pixel distribution is more uniform, which proves that the algorithm is more secure.

### 6.5. Correlation Analysis

**Figure 17:**
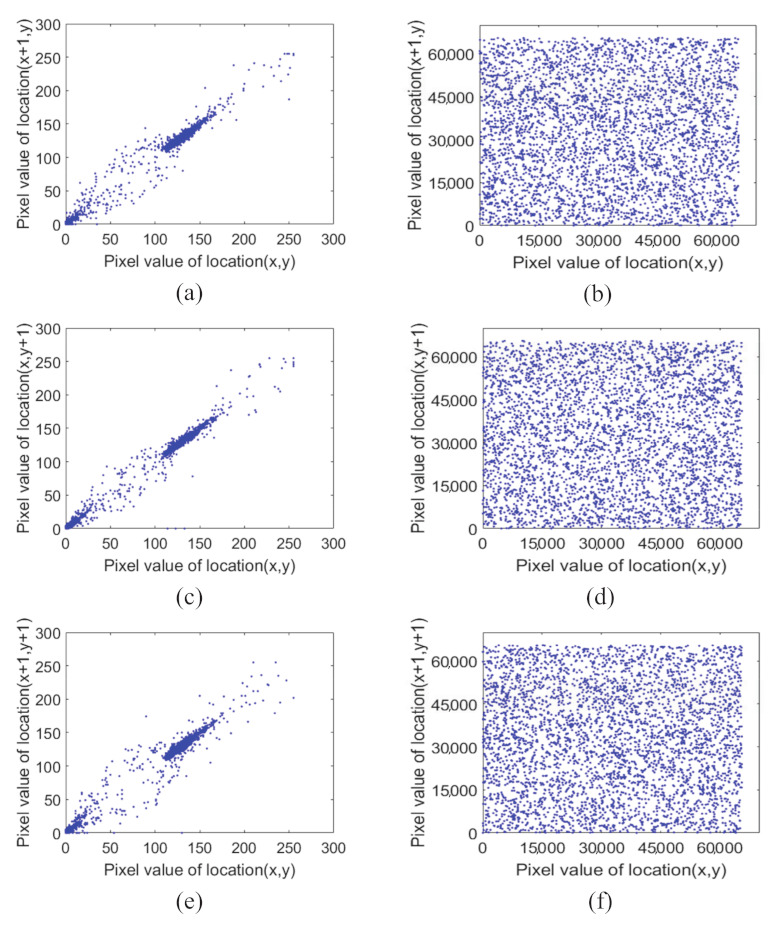
Correlation diagram of two adjacent pixels of CT Abdomen: (**a**,**c**,**e**) Correlation diagram of adjacent pixels in horizontal, vertical and diagonal directions of plaintext image; (**b**,**d**,**f**) Correlation diagram of adjacent pixels in horizontal, vertical and diagonal directions of ciphertext image.

There are a lot of data in the plaintext image, but there is also the issue of data redundancy, which leads to a strong correlation between its neighboring pixels. The correlation coefficient is a statistical indicator that reflects the degree of close correlation between variables and can be used to represent the connection between pixels. The correlation coefficient is used in image encryption security analysis to test the correlation between adjacent pixels, and the closer the correlation coefficient value is to 1, the greater the degree of correlation between two variables. When the value approaches zero, the two variables are considered uncorrelated. To secure data from various attacks, an effective encryption scheme should disrupt this link between neighboring pixels. A total of 5000 pairs of adjacent pixels were randomly selected from the plaintext and ciphertext images of “Abdominal CT”, and the correlation coefficients were calculated from the horizontal, vertical, and diagonal directions, respectively, by the following equation [48].

(29) $$\left\{\begin{array}{c} \;\;\;\;\;\;\;\;\;\;\;\;\;\;\;\;\;\;\;\;\;\;\;\;\;\;\;\;\;\;\;\;\;\;\;\;\;\;corr\left(x,y\right)=\frac{cov(x,y)}{\sqrt{D(x)D(y)}},\\ cov\left(x,y\right)=\frac{1}{N}\sum_{i=1}^{N}\left(x_{i}-E\left(x\right)\right)\left(y_{i}-E\left(y\right)\right),\\ \;\;\;\;\;\;\;\;\;\;\;\;\;\;\;\;\;\;\;\;\;\;\;\;\;\;\;\;\;\;\;\;\;\;\;\;\;\;\;\;\;\;\;\;\;E\left(x\right)=\frac{1}{N}\sum_{i=1}^{N}x_{i}.\\ \;\;\;\;\;\;\;\;\;\;\;\;\;\;\;\;\;\;\;\;\;\;\;\;\;\;D\left(x\right)=\frac{1}{N}\sum_{i=1}^{N}{\left(x_{i}-E\left(x\right)\right)}^{2}, \end{array}\right.$$

where *x* and *y* are the neighboring pixel values in an image. Figure 17 depicts the results, and Table 2 depicts the correlation coefficients, which show that the correlation coefficients of the cryptographic images are approximately equal to 0. This means that, in the horizontal, vertical, and diagonal directions, adjacent pixels in cryptographic images have very little correlation. Consequently, the suggested encryption scheme is resistant to pixel correlation statistical assaults.

### 6.6. Information Entropy Analysis

Information entropy is a metric for quantifying information and determining the amount of data required. The randomness of picture information in an image is represented by information entropy, which is a key indication for testing the performance of encryption techniques. Information entropy describes the chaos of image information, and the higher the information entropy, the more disordered the image information is. On the other hand, the lower the information entropy, the more ordered the visual data is. The ideal amount of information entropy for an 8-bit cipher image is 8. Similarly, if the cipher image pixels are represented by 16 bits and the cipher picture’s ideal value is 16, the information entropy of the image encrypted by our approach should be close to the ideal value of 16 as we convert the cipher image to 16-bit pixels. Table 3 illustrates the test results, indicating that the information entropy is approaching the optimal value. The following equation can be used to determine the information entropy [36].

(30) $$H\left(c\right)=\sum_{i=0}^{65535}p\left(c_{i}\right)\log_{2}\frac{1}{p\left(c_{i}\right)}$$

where ($c_{i}$) denotes the pixel value of the image and $p(c_{i})$ is the probability of pixel occurrence.

### 6.7. Differential Attacks Analysis

The differential attack is a crucial test for evaluating image encryption methods. Its fundamental method is to encrypt the plaintext image by changing the pixel value and then attack the encryption technique by comparing it to the original encrypted image. As a result, a good picture encryption method should be resistant to differential attacks. The uniform average change rate (UACI) and the number of pixel change rate (NPCR) are two measures used to assess encryption systems [52].

Where *M* is the image length, *N* is the image width, and $C_{1}$ and $C_{2}$ are the two ciphertext images before and after the original plaintext image is changed by one pixel value. For a 16-bit grayscale image, the ideal value of UACI is about 33.33333% of the bit, while the ideal value of NPCR is about 99.9985%. We tested the NPCR and UACI for different images, and the test results are shown in Table 4. The experimental results show that the present encryption scheme has better robustness against differential attacks. In addition, the results also demonstrate that the present encryption algorithm has high plaintext sensitivity.

### 6.8. Select Plaintext/Ciphertext Attack Analysis

To resist selective plaintext/ciphertext attacks, the key should be made to contain information related to the plaintext [49]. In generating chaotic sequences, part of the key used in this paper is obtained from the label information in the plaintext image, which means that if different label information is selected, then the key will also change, indicating that the ciphertext image is more dependent on the plaintext image and, therefore, our proposed algorithm is capable of effectively resisting both known plaintext and selected plaintext attacks.

### 6.9. Robustness Analysis

**Figure 18:**
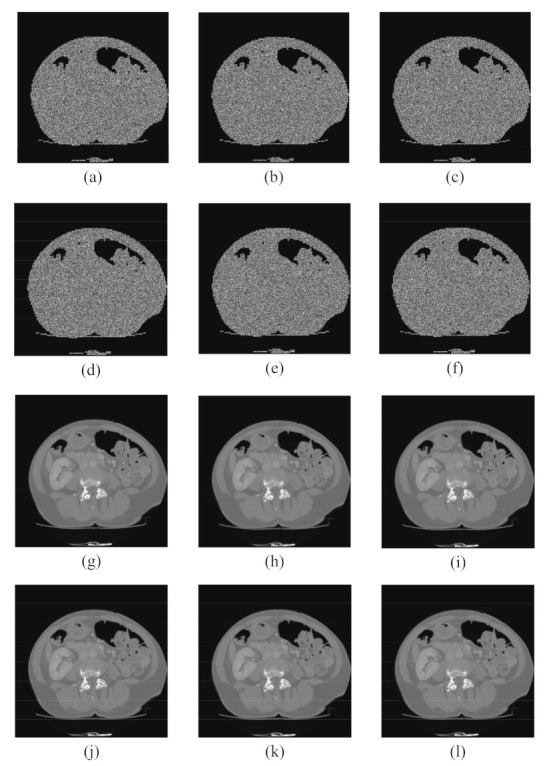
Noise attack results: (**a**) Ciphertext image after SPN attack, noise density = 0.000001; (**b**) Ciphertext image after SPN attack, noise density = 0.00001; (**c**) Ciphertext image after SPN attack, noise variance = 0.00005; (**d**) Ciphertext image after SN attack, noise variance = 0.000001; (**e**) Ciphertext image after SN attack, noise variance = 0.000003; (**f**) Ciphertext image after SN attack, noise variance = 0.000005; and (**g**–**l**) are the decrypted images corresponding to (**a**–**f**).

Robustness analysis is used to evaluate the resistance of an image to data changes or loss during transmission [53]. Images may be affected by noise during transmission, and these noise attacks may cause the images not to be decrypted properly. We used two different levels of noise attacks on ciphertext images, namely, salt-and-pepper noise, and scattered noise. The experimental results are shown in Figure 18. To quantitatively measure the reliability of defense against noise and data loss, we compared the PSNR of the original and decrypted images. The peak signal-to-noise ratio (PSNR) is a mathematical estimate of image quality between the original and noise-added images. The higher the PSNR number, the closer the original and decrypted images are. The calculation formula of PSNR is shown in Formula (31) [53]:

(31) $$\left\{\begin{array}{c} MSE=\frac{\sum_{i=1}^{M}\sum_{j=1}^{N}{\left(P\left(i,j\right)-C\left(i,j\right)\right)}^{2}}{M\times N},\\ \;\;PSNR=10\times log_{10}\left(\frac{MAX^{2}}{MSE}\right), \end{array}\right.$$

where MSE denotes the mean square error between the original image *P* and the encrypted image *C*. MAX denotes the maximum pixel value of the image, MAX = 256 for 8-bit images and MAX = 65,536 for 16-bit images. *P* is the original image and *C* is the decrypted image after adding noise. Table 5 displays the test results and the PSNR is more than 44 dB, as can be seen, which indicates that the algorithm is resistant to noise attacks and has a high level of robustness.

In addition to noise attack, clipping attack is also a common attack method. In our proposed algorithm, instead of encrypting the whole medical image, the medical image’s ROI is encrypted. The ROI location information buried in the ciphertext image will be lost if the ciphertext image is clipped, rendering the ciphertext image unrecoverable.

It should be noted, in particular, that medical images contain critical patient pathology information. Even minor tampering or data loss can have a significant impact on a doctor’s diagnosis. As a result, one should try to avoid causing image damage (such as noise or clipping attacks) or implement appropriate warning policies after such attacks and re-upload the image to ensure the integrity of the image information and the physician’s access to the entire medical image.

### 6.10. Speed Analysis

The encryption speed is a significant indicator in the process of practical application, in addition to assessing the security of the encryption method. We completed the simulation tests on MATLAB 2015b platform with Intel Core i3 1.70 GHz CPU and 8 GB RAM. For comparison, full image encryption and region of interest encryption were performed on medical images of size 512 × 512 and 320 × 320, respectively, with the encryption scheme proposed in this paper. The encryption scheme’s efficiency is determined by the encryption time (ET) and throughput (ETP) [54]. These computing statistics are expressed as follows:

(32) $$ETP=\frac{Imagesize}{Encryptiontime(sec)}$$

For the above test images, Table 6 shows the encryption time, throughput, and the number of cycles per byte required. As may be shown, the proposed encryption method’s experimental results are superior, with a very short encryption time and high throughput, which can meet the applicability in real-time security applications. Compared with other encryption algorithms, our proposed encryption algorithm can meet the requirements of fast encryption.

We should point out that the complexity of the algorithm is to measure a key element of the efficiency of encryption in the encryption algorithm we proposed; the algorithm’s time complexity with the size of the area of interest. This is related to the amount of interested area in that the more interest in regional elements, the higher the time complexity of algorithm, and the encryption time will also increase.Table 6 provides the results of the encryption time, ROI amount, throughput, and number of cycles per byte required for the encryption process for the above test images. We can see that the proposed encryption method has good experimental results, short encryption time, and high throughput, which can meet the applicability of real-time security applications. Compared with other encryption algorithms, our proposed encryption algorithm can meet the requirements of fast encryption.

## 7. Conclusions

The following issues are looked into in this paper. The first proposal is for a new type of one-dimensional chaos. The system has a greater parameter range and better chaotic behavior than the existing Logistic and Sinusoidal mapping systems. It is proven that the 1D-ECC system can generate good chaotic sequences using the bifurcation diagram, Lyapunov exponent, information entropy, and 0–1 test. Secondly, we devise a new key generation approach based on the DICOM label information of medical images, in which the key is used to launch a new chaotic mapping to generate chaotic sequences, resulting in a key that is highly related to the plaintext image. On this foundation, a region of interest (ROI) encryption algorithm for medical images that uses helical scanning scrambling and bidirectional diffusion is created. The algorithm we proposed has a good encryption effect and can withstand many known assaults, according to simulation results and security analysis. Finally, in future work, we will consider combining with asymmetric algorithms to solve the security risks of key transmission, further improve the security of medical images, and guarantee the accurate and efficient transmission of medical images.

## Figures and Tables

**Table 1:** NPCR and UACI of ciphertext images generated by correct key and wrong keys.

Test	Key1	Key2	Key3	Key4	Key5	Key6
NPCR (%)	99.9985	99.9985	99.9938	99.9977	99.9977	99.9969
UACI (%)	33.3528	33.3331	33.1994	33.3201	33.3564	31.8352

**Table 2:** Correlation coefficients of ciphertext images.

Image	Horizontal	Vertical	Diagonal
MRI Brain	−0.0077	−0.0030	0.0004
CT Brain	−0.0001	−0.0097	−0.0038
CT Abdomen	0.0009	−0.0010	−0.0013
CT Lung	−0.0080	0.0010	−0.0074
Ref. [49]	0.0193	−0.0154	0.0032
Ref. [50]	−0.0394	−0.0194	−0.0223

**Table 3:** Information entropy test.

Image	Image Size	Plaintext Images	Ciphertext Images	Ciphertext Image (8 bits)
MRI Brain	320 × 320	6.3125	15.0135	7.9981
CT Brain	512 × 512	6.3125	15.6177	7.9993
CT Abdomen	512 × 512	6.3125	15.3090	7.9993
CT Lung	512 × 512	6.3125	15.5872	7.9992
Ref. [36]	512 × 512	6.3125	15.3083	7.9992
Ref. [4]	512 × 512	6.3125	-	7.9989
Ref. [51]	512 × 512	6.3125	-	7.9986

**Table 4:** NPCR and UACI.

Test	CT Abdomen	CT Lung	CT Brain	MRI Brain
NPCR (%)	99.9985	99.9974	99.9983	99.9982
UACI (%)	33.3281	33.2819	33.3566	33.2931

**Table 5:** PSNR of decrypted image and original image.

Noise Attack	PSNR
SPN 0.000001	+∞
SPN 0.00001	50.9532
SPN 0.00005	45.3259
SN 0.000001	+∞
SN 0.000003	65.2320
SN 0.000005	44.7703

**Table 6:** Encryption time and efficiency.

Image	Image Size	Full Encryption	ROI Encryption	t	The Number of ROI	Encryption Throughput
MRI Brain	320 × 320	0.141 s	0.015 s	55	54,144	55.08
CT Lung	512 × 512	0.190 s	0.023 s	53	78,656	52.18
CT Abdomen	512 × 512	0.189 s	0.037 s	66	130,336	53.75
CT Brain	512 × 512	0.181 s	0.031 s	45	116,784	57.48
Ref. [36]	512 × 512	0.186 s	0.120 s	70	39,168	4.98
Ref. [36]	320 × 320	0.186 s	0.120 s	70	39,168	4.98
Ref. [54]	256 × 256	0.010 s	-	-	-	50
Ref. [55]	512 × 512	0.287 s	-	-	-	6.9
Ref. [56]	512 × 512	1.26 s	-	-	-	1.58

## Data Availability

The used test images are all included in the paper.
